# Goals as Emergent Autopoietic Processes

**DOI:** 10.3389/fbioe.2021.720652

**Published:** 2021-11-19

**Authors:** Tomas Veloz

**Affiliations:** ^1^ Centre Leo Apostel for Interdisciplinary Studies, Vrije Universiteit Brussel, Brussels, Belgium; ^2^ Fundacion para el Desarrollo Interdisciplinario de la Ciencia, la Tecnologia y las Artes, Santiago, Chile; ^3^ Universidad Andres Bello, Facultad de Ciencias para la Vida, Santiago, Chile

**Keywords:** chemical organization theory, emergence, goals, autopoiesis, process modelling

## Abstract

While the phenomena of reaching a goal is generally represented in the framework of optimization, the phenomena of becoming of a goal is more similar to a “self-organization and emergent” rather than an “optimization and preexisting” process. In this article we provide a modeling framework for the former alternative by representing goals as emergent autopoietic structures. In order to conceptually situate our approach, we first review some of the most remarkable attempts to formally define emergence, and identify that in most cases such definitions rely on a preexisting system to be observed prior and post emergence, being thus inadequate for a formalization of emergent goals corresponding to the becoming of a systems as such (e.g. emergence of life). Next, we review how an implementation of the reaction networks framework, known as Chemical Organization Theory (COT), can be applied to formalize autopoietic structures, providing a basis to operationalize goals as an emergent process. We next revisit the definitions of emergence under the light of our approach, and demonstrate that recent taxonomies developed to classify different forms of emergence can be naturally deduced from recent work aimed to explain the kinds of changes of the organizational structure of a reaction network.

## 1 Introduction

How can it be that simple rules of evolution at the individual level provide complex properties at a global level? Consider for example a game like chess, where the rules of the game are very simple to understand, and both the evolution of the game and the goal of it are perfectly defined by particular state configurations and transitions.

However, in order to be a good player (or in order to understand the intentions of a good player) it is necessary to not only implement configurations and transitions in our mind, but also determining which sequences of transitions drive to a winning result. The latter is an optimization process that requires an exponential amount of computation with respect to the number of movements ahead the player is willing to implement. Such optimization capacity, even for moderately large sequences of moves, cannot be directly implemented by humans. Instead, expert human players rely on concepts they create to describe the *higher level properties of the game*, so the space of possibilities can be explored in a more abstract and simpler representation of the game. These higher level properties do not describe the actual state of the game, but relational properties among the pieces which can be considered as (dis)advantageous to the player. For expert players thus, the goal of the game shifts from reaching a winning state, to reaching certain advantageous higher level relational properties, which with high confidence will unfold in a winning state.

These higher-level properties can be thought of as emergent goals, subjected to the larger winning goal, and cannot be explained in terms of the direct interactions of the entities composing the system, but as global dynamical relations. Thus, emergent properties are considered the “key ingredient that makes complex system complex” (Deguet et al., 2006). Notably, several emergent goals do not (at least directly) comply with a previously understood major goal. An example of this kind of goal correspond to major transitions in evolution such as the emergence of life or the emergence of language (Varela, 1979), where there is powerful evidence that the idea of system, expressed as chemical reactions and communication events respectively, suffers a radical change after the transition.

In order to comprehend the notion of emergent goal we must rely on sensible definitions of both emergence and goal. Goals are generally understood as an idea of the future or desired result that a person or a group of people envision, plan and commit to achieve (Locke and Latham, 1990). This line of thinking has been applied mostly to organizational and behavioural psychology, and incorporate various interesting systemic and evolutionary concepts such as resilience, adaptation, and of course emergence. However, these approaches lack of a formal framework from which models can be scientifically linked to experimental observations (Locke and Latham, 2002). Other attempts to explaining what a goal is, more related to teleology, teleonomy, and purpose, instead of starting from a priori envisioned goals, rely on the formalism of agents-based modeling or neural networks to establish a notion of goal as desired properties to be obtained in future states of their dynamics (Atlan, 1998; Louzoun and Atlan, 2007). However, several of these approaches rely on the properties of particular states as the defining element of what a goal is, and thus neglect non-static features which might not be properties of the states themselves, but relational properties among the states that the system visits (Goguen and Varela, 1979).

One important example of such non-static kind of goal is life. Living systems are such not because they reach states having certain properties, but because of their ability to self-maintain their structure under different circumstances. Hence, the capacity for a system to reach the goal of being alive is not in its states, but in its ability to perform processes that ensure the maintainance of its self-productive structure.

Regarding emergence, there are important examples that are generally used to explain its meaning (de Haan, 2006; wikipedia). We describe four of these examples to clarify what is commonly understood as emergence:

• Flocks of birds: *hundreds of birds flying in amazing synchronicity cannot be explained from the simple rules which follow every single bird; keeping some distance while stay aligned with its nearest neighbours and avoid predators.*
• Colony of ants: *Ants have simple rules of behaviour from the pheromone trail that can breath and the environment observations, but the ant colony exhibits a notable capability of organisation, exploring and exploiting their surroundings, and even deciding as a group the place in which they are going to made the colony.*
• Friction: *Forces between elementary particles are conservative. However, friction emerges when considering more complex structures of matter, whose surfaces can convert mechanical energy into heat energy when rubbed against each other. Similar considerations apply to other emergent concepts in continuum mechanics such as viscosity, elasticity, tensile strength, etc.*
• Stock market: *The purely self-interested actions of thousands of buyers and sellers results in complex global characteristics of economy such as shifts in activity and valuation or bubbles and crashes.*

There are various approaches to define emergence in philosophy and in various branches of science (Mill, 1843; Darley, 1994; Bonabeau and Desalles, 1997; Holland, 1998; Johnson, 2001; Keith Sawyer, 2001; Kubík, 2003; Hauptman, 2007). Most accepted approaches to emergence rely on the fact that emergent properties cannot be directly deduced from a local point of view, but require the evolution (unfolding) of the system in a larger time-scale than the scale at which local interactions are defined, and also requires the capacity to observe the system at a global scale (in space or quantity), beyond the scale (in space or quantity) of local interactions.

Therefore, emergence is a concept that does not reside in the state of the entities, but in their space-time (and other qualia) dynamics, and in the properties of the relations driving such dynamics. Hence, we propose that emergent goals (such as life) are better explained in terms of the relational processes that constitute their dynamics of existence rather than in terms of the states they reach and the properties that can be observed for such states (Rubin et al., 2021).

Among the definitions of emergence, while most definitions attempt to differentiate two layers of description (or observation), one specifying the *rules of the entities forming the system*, and another specifying the *behaviour of the system as a whole*, a small proportion of such definitions attempts at formalizing what these two layers contain, and on what operational basis these two layers can be linked. Additionally, several definitions of emergence lack of a formal definition of what a system is and what is to observe a system. Thus, a formalization of the notion of emergence where all concepts are developed in terms of objects and operations among these objects, i.e. an operationalization of the notion of emergence, would permit its proper measurement and quantification, and in this sense we could subject the concept of emergence to unambiguous scientific enquiry (for a review of the notion of operationalization of systems and its relation to synthetic biology we refer to (Wolkenhauer, 2001)). Such approach will be equally useful to synthetic biologists studying emergence of life and other major transitions in evolution which set up their own goal, as well as to other domains of basic and social scientists attempting to explain goals that become such *via* emergent processes.

In this article we operationalize the notion of goal by first proposing an operational notion of system, and then of goal as an emergent process within it. In particular, we propose that process based modeling, and particularly chemical organization theory (COT) allows to characterize the notion of system and the phenomenon of goal emergence in a clear way. In previous work we have developed an operational account of the notion of system using reaction networks (Veloz and Razeto-Barry, 2017a) and have shown that organizations (in the COT sense), corresponding to collections of species in a reaction network that form closed and self-maintaining sub-networks, can model systems of not only (synthetic) biological, but of diverse (and even interdisciplinary) nature (Veloz et al., 2014; Veloz, 2020; Veloz and Flores, 2021).

Organizations implement an abstract form of autopoietic system, as they are able to persist in time through their operationally closed processes of self-production (Dittrich and Di Fenizio, 2007; Veloz et al., 2011). Although organizations have no reference to the spatial configuration of the entities involved at the spatial level, they represent all forms of self-productive dynamics of a reaction network (Centler et al., 2008; Gruenert et al., 2010). We propose that organizations play the role of potentially emergent systems, and that the feedback control mechanisms that allow for the self-productive processes of a particular organization represent its goal-orientated behavior. Moreover, we show the different kinds of emergent properties described in the literature (Fromm, 2005) can be operationally described in terms of different kinds of changes that a reaction network can undergo, i.e. changes beyond the level of states, as studied in traditional dynamical systems sense, incorporating also changes on the dynamical rules as well as at the topological level, recently introduced in (Veloz and Razeto-Barry, 2017b; Busseniers et al., 2021; Veloz et al., 2021).

In section 2 we provide a critical review of the most remarkable attempts to operationally define emergence, and of the types of emergent properties proposed in the literature. In section 3 we introduce the COT formalism and propose that organizations provide an operational framework of goals as emergent processes, and that the different types of goals presented in the literature can be mapped to the different types of change that a reaction network can undergo. Next we summarize our approach and conclude proposing future lines of application.

## 2 Emergence

In this section we provide a critical review of remarkable attempts to provide a formal notion of emergence. We focus on the approaches that have attempted to develop an operational formalization of the concepts applied to define emergence. Therefore, definitions of emergence which rely on concepts at the natural language level, and do not provide a way to link such concepts to operational structures are left out. To the knowledge of the authors, we provide a comprehensive account of the attempts to operationalize the concept of emergence.

For comprehensive reviews of the notion of emergence covering both operational and non-operational definitions, as well as taxonomies of emergence, we recommend (Deguet et al., 2006) and (Fromm, 2005) respectively.

### 2.1 Bonabeau and Desalles Definition

*Emergence can be defined to take place at the moment when some detector finds some new feature that makes the overall description of the system simpler than it was before* (Bonabeau and Desalles, 1997).

In order to formalize the definition, they consider a detector to be any device which gives a binary response to its input. Relative complexity *C*(*S*|*D*, *T*) of a system *S*, where *D* is a set of detectors and *T* a set of available tools that allow to compute description of structures detected through *D*. Emergence happens when between time *t* and *t* + Δ*t*, two events happen:

1. A detector *D*_*k*_ becomes activated.
2. *C*_*t*+Δ*t*_(*S*|*T*, *D*_1_, …, *D*_*k*_) < *C*_*t*_(*S*|*T*, *D*_1_, …, *D*_*k*−1_).

Bonabeau and Desalles define emergence supported by concepts of time, detection and relative complexity. It is important to note that Bonabeau and Desalles quantifies the emergence from the reduction of complexity of the system’s description.

Bonabeau and Desalles definition of emergence does not provide a clear framework because it is not clearly operationalized what a *system* is, what are the *tools that allows to compute the structures*, what are the *detectors* and how the notion of simpler is operationalized from the previous notions. Therefore, the formalization of the definition is incomplete.

### 2.2 Ronald and Sipper Definition

*Languages are used to describe a system. A designer may use one language* *L*_1_ *to describe the interactions between some set of basic elements, yet another distinct language (* *L*_2_ *) may turn out to be more useful in describing their overall behaviour. This generally happens because* *L*_2_ *has terms that refer to coherent entities which have no name in the lower language* *L*_1_ *. So the emergent process makes the new language both necessary and useful* (Ronald et al., 1999).

The latter idea of emergence is framed in a test of emergence:

• *Design*: The system has been constructed by the designer by describing local elementary interactions between components in a language *L*_1_.
• *Observation*: The observer is fully aware of the design, but describes global behaviour and properties of the running system, over a period of time, using a language *L*_2_.
• *Surprise*: The language of design *L*_1_ and the language of observation *L*_2_ are distinct, and the causal link between the elementary interactions programmed in *L*_1_ and the behaviours observed in *L*_2_ is non-obvious to the observer, who therefore experiences surprise.

Ronald and Sipper definition was proposed in the context of artificial life. They gave an *emergence tag* to a wide number of artificial life phenomena which accomplish the requisites of emergence test. However, their examples are not rigorously treated.

The latter definition relies on the differences between languages as the principal feature of emergence recognition, which can in principle be formalized. However, the definition lacks an operational specification of what a system is, or a formal definition of language. Indeed, since formal languages exist we could constrain ourselves to a formal representation such as for example logic in the sense of model theory (Ebbinghaus and Flum, 1999). However, in such a case we would need to properly define what a difference between languages is, so a surprise can be properly identified.

### 2.3 Nils Baas Definition

A notable attempt to formalize emergence is due to Baas (Baas and Emmeche, 1997). He argues that emergent properties refer to the observer, instead of the system which is observed. The framework proposed by Baas is the following:

Let *S* be a system composed by a family of agents, Obs be a set of observation mechanisms and Int be a set of interactions between agents.

Observation mechanisms measure properties on which the interactions might depend. Interactions among agents over time generate a new system of agents:*S* ^2^ = *R*(*S*, Obs, Int)

Which is the result of the interactions. This could be a stable pattern or a dynamically interacting system. We call *S* ^2^ an emergent structure which may be subjected to new observational mechanisms Obs′. This leads to:

Definition

*P* is an emergent property ⇔ *P* ∈ Obs′(*S* ^2^) and *P*∉Obs′(*S*).

The observational mechanism may be internal or external to the family of agents.

Baas purposes the above definition and also gives a number of examples (in physics and mathematics principally) in which his definition is applicable. The definition is based in the notion of observations, interactions, and properties, which are already operationalized in the examples treated by the author. However, when trying to explore emergence in domains where such notions are not properly formalized the application of this definition becomes difficult.

### 2.4 Vince Darley Definition

*A phenomenon is emergent if the amount of computation,* *s*(*n*)*, required to produce it cannot be reduced by any deeper understanding or shortcuts of any kind* (Darley, 1994)

In this approach, Darley proposes a formal tool to compare properties: how much computation is needed for verifying a property given a system with *n* agents. Therefore, we consider this approach to emergence fully operational because the entities required in the definition of emergence are mapped to entities that can be computed and thus can be formally defined.

He proposes two scenarios: The first as he called *deeper understanding*, is the knowledge that we have for expressing properties (called *symmetries* in his approach) of the system, and it is assumed to need an amount of computation *u*(*n*) to be produced. The second is *s*(*n*), the optimal amount of computation needed by a simulation to reproduce the property. If *u*(*n*) ≥ *s*(*n*), then the property is emergent.

For Darley, the amount of computation is the measure of complexity of the property to be measured in the system, Since his comparison is quantitative, then there are properties *more emergent* than others, in fact he defines an emergence ratio $\frac{u\left(n\right)}{s\left(n\right)}$ to characterize the *emergence* of the property.

This definition is interesting, but it is vague in some respects. First, the notion of *deeper understanding* is ad-hoc as it relies in the definition of symmetries of the system, which will vary in representation according to the kind of system being studied. Second, the notion of *optimal simulation* is not clearly defined. Indeed, the author refers to it as *God’s simulation*, which is useful from a conceptual perspective, but not for scientific purposes.

### 2.5 Aleš Kubík Definition

Let us consider a multi agent system composed of the environment and some agents. Basic emergence then refers to a property of the system that can be produced by interactions of its agents (components) with each other and with the environment and cannot be produced by summing behaviors of individual agents in the environment (Kubík, 2003).

Kubík definition of emergence relies on the language to a multiagent systems (MAS), and developed within the framework of grammars. Grammars in this context can be used to represent properties of agents by means of the vocabulary, words refer to agents or environmental configurations of properties, and grammar rules reflect the possible interactions occurring in the model (Csuhaj-Varjú et al., 2018). In this sense this framework is fully operational because the system and the measurement of properties is formally defined.

Indeed, the notion of emergence is inspired in the idea that different grammars, when writing in the same tape, can produce words and languages that cannot be obtained by any of the grammars in isolation.

Since MAS can be represented by grammars, he formalizes the behaviour of agents as grammars and define a *superimposition operation* over the agents’ languages which can be interpreted as *the sum of conditions the agents can bring about in the environment if they act individually in the environment*. If there exist a word in the language of the MAS system which is not in the superimposition of the languages of the agents, then the grammar system has the property of *basic emergence*.

One drawback of this definition is that the superimposition operation might be extremely hard to compute, as it relies on the reachability of words which is known to be a computationally hard problem (Schmitz, 2016). Moreover, some emergent behaviour can be identified within the superimposition operator. For example, the ability of a cell to self-produce is not beyond the actions that its components perform. It only requires the proper activation of the metabolic pathways so the self-production is reached.

### 2.6 Taxonomies of Emergence and the Four Types of Roles

Interestingly, different kinds of emergence have been defined for systems with more complex features such as changing environment or evolutionary interactions. Those complexifications about the way the system is defined have been utilized to define taxonomies of forms of emergence. Fromm in (Fromm, 2005) has summarized the various taxonomies developed by big figures in science and philosophy such as David Chalmers (Chalmers, 2002), Stephen Wolfram (Wolfram, 1984), and Yaneer Bar-Yam (Bar-Yam, 2004). In addition, he proposes its own taxonomy encompassing the other taxonomies, by considering four classes which can implement different kinds of feedback mechanisms. He explains his taxonomy in a more intuitive way as follows:

The classification (of emergence) can also be seen from a different perspective, if it is specified in terms of constrained generating processes or roles: type I corresponds to fixed roles, type II to flexible roles, type III to the appearance of new roles and the disappearance of old ones, type IV to the opening of a whole new world of new roles. Another classification possibility is to use different levels of prediction: intentional emergence of type I is predictable, weak emergence of type II is predictable in principle (though not in every detail), multiple emergence of type III is chaotic or not predictable at all, strong emergence of type IV is not predictable in principle.

We summarize the two emergence taxonomies in Table 1.

**TABLE 1:** Summarizing types of emergent properties in terms of both role modifications of the entities forming the system and predictability.

Type	Role evolution	Dynamical prediction	Structural change
I	Fixed	Full	Null
II	Constrained change	Major	Null
III	Unconstrained moderate change	Minor	Minor
IV	Unconstrained radical change	Null	Major

Fromm’s taxonomy can be better understood in terms of how the unfolding dynamics of the system, included the changes of the rules and entities involved in it, drive to emergent properties where the roles of the entities might be slightly to completely different from the roles they can play in the fixed dynamics, i.e. when rules are not added or eliminated and applied the same way.

Namely, when roles remain fixed, the unfolding dynamics can be predicted and thus we are in a situation where the structure of the system does not suffer any modification. When roles can change within certain constrains, the dynamical prediction becomes reduced by the flexibility of the rules (e.g. stochastic dynamics), but the structures underlying the description of the model remain unchanged. When roles are unconstrained in their change, but the changes are moderate, we have a very limited capacity to predict the dynamics of a given initial state because the structure is constantly changing, indeed, the dynamics in the traditional sense of dynamical systems is unpredictable, as the state might change chaotically. However, at an abstract level we might still identify regions of the phase space which are active, e.g. identifying which agents will be active without knowing in which way. When roles change in unconstrained and radical way, we loose the capacity to predict the dynamics of the model not only at the state but also at the structural level because the information with which the model begins becomes completely different as the dynamics unfolds.

We will show in the coming section that this classification can be directly formalized in terms of reaction network processes.

## 3 Reaction Networks, COT and Emergence

**FIGURE 1:**
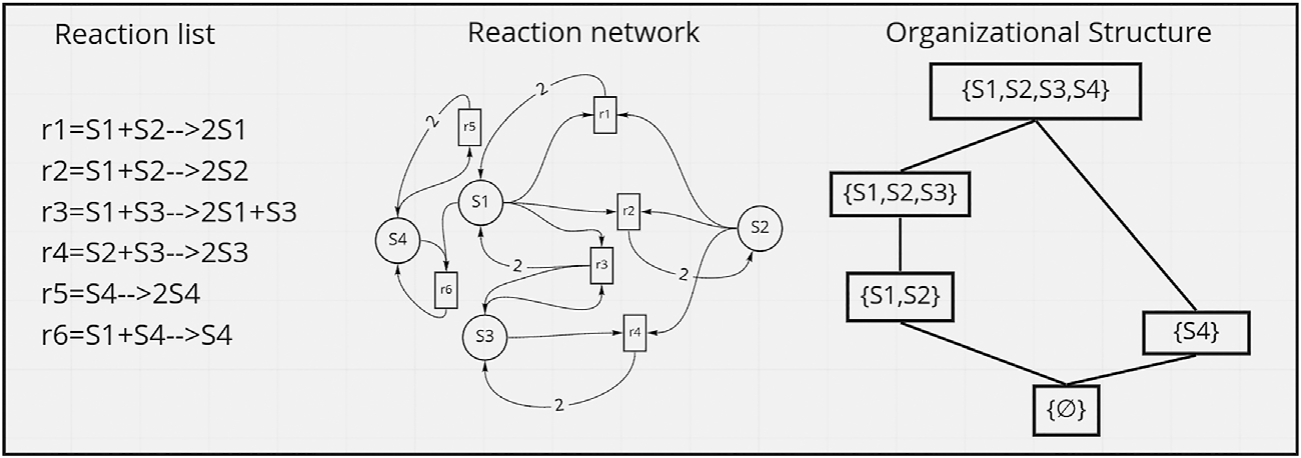
Example of a reactions network, and its induced hierarchy of organizations. Inspired from (Veloz, 2020).

A reaction network is defined by a pair (M,R) , where M={a,b,…} is a set of molecular species, and $R\subseteq P_{m}\left(M\right)\times P_{m}\left(M\right)$ is a set of reactions, where $P_{m}\left(M\right)$ denotes the set of multisets of M . For example, in the reaction network of Figure 1, reaction *r*_5_ = *s*_4_ → *s*_4_ represents a self-reproduction process of species *s*_4_, reaction *r*_6_ = *s*_1_ + *s*_4_ → *s*_4_ represents the destruction of species *s*_1_ out of the interaction of species *s*_1_ and *s*_4_, and *r*_3_ = *s*_1_ + *s*_3_ → 2*s*_1_ + *s*_3_ represents the reproduction of species *s*_1_ catalized by *s*_3_.

Recently, reaction networks have been proposed as a framework to represent systems of diverse, and even interdisciplinary, nature (Veloz and Razeto-Barry, 2017a). Species play the role of the fundamental interacting entities. These can be not only of physical, but of cognitive, memetic, or cultural nature. Reactions play the role of transformative processes, which change certain collections of species into other collections of species. Interestingly, the transformation pathways that can be built upon the set of reactions allow to define *organizations*, which correspond to sub-networks under which pathways that leave invariant the structure can occur. When these pathways can be reached and fixated from a certain initial condition, we (externally) observe that the reaction network *self-organizes*. Indeed, Dittrich in (Dittrich and Di Fenizio, 2007) developed Chemical Organization Theory (COT) upon a theorem showing that for every fixed point of a reaction network, its active part, i.e. the species with concentration higher than a minimum (concentration threshold) value, form a sub-network that is an organization.

Therefore, the reaction network (M,R) is not intended to represent a priori a system in this approach, but a universe of possible interactions. Systems correspond to sub-networks that are persistent enough to be observed as an individual unit over time, i.e. organizations (Veloz and Razeto-Barry, 2017a).

Interestingly, organizations form a hierarchy of sub-networks in the reaction network that represent all possible structures that can be persistent in time, and thus can be considered as the *emergent systems* that can possibly be observed in a time-scale which is much larger than the time-scale of the interactions (Veloz and Razeto-Barry, 2017a) (*see* Figure 1 right).

There are various mathematical properties that on the one hand facilitate their computation and on the other hand provide conceptual tools to understand the inner workings of self-organization. We will not dig in the details of the theory, but refer the interested reader to (Veloz and Razeto-Barry, 2017b; Veloz et al., 2018) and references therein.

COT complements a solid and actively growing theoretical framework to study the dynamics of a reaction networks at both quantitative and qualitative level. Reaction networks can be studied using difference, stochastic or differential equations and a vast amount of literature is devoted to complement these approaches with structural analysis of the network (Fell, 1992; Wilkinson, 2006).

### 3.1 Closed Reaction Networks

Since COT concerns with the sub-networks of a reaction network, it is relevant to establish some notation to provide clear definitions. For each r∈R , let supp(*r*) to be the set of reactants of *r*, and let prod(*r*) the set of products of *r*.

Consider a set of species X⊆M . Since not all reactions are such that supp(*r*) ⊆ *X*, we define $R_{X}$ as the set of reactions r∈R such that supp(*r*) ⊆ *X*, and is named the set of reactions associated to *X*. Therefore, $\left(X,R_{X}\right)$ is a sub-network of (M,R) associated to *X*.

For notational simplicity we extend the support and product definitions as follows

$$supp\left(R_{X}\right)=\cup_{r\in R_{X}}supp\left(r\right),\;prod\left(R_{X}\right)=\cup_{r\in R_{X}}prod\left(r\right).$$

A crucial feature in COT is the notion of closed set. We say a set *X* is closed if and only if $prod\left(R_{X}\right)\subseteq X$ . The defining feature of closed sets is that there is no qualitative novelty in their dynamics. Therefore, it is not complicated to deduce that the long term dynamics of a (finite) reaction network tends to a closed set.

Closed sets form a hierarchy within the reaction network that can be embedded in a lattice. From here, several computational and structural properties have been developed in recent years. We will not dig further on the structural properties of closed sets but refer to (Veloz et al., 2018) for further analysis.

### 3.2 Processes, Autopoiesis and Organizations

In order to comprehend the dynamics of a reaction network, we must equip the reaction network with a way to calculate the occurrence of the reactions. A *process* represents the occurrence of reactions over an interval of time. Thus, a process specifies a *collective transformation of species* in the reaction network over a timeframe (Veloz and Razeto-Barry, 2017a).

The stoichiometric matrix **S** encodes how many species are consumed and produced by all the reactions in the rection network (M,R) . Given a set X⊆M we denote the reduced stoichiometric matrix of the subnetwork $\left(X,R_{X}\right)$ by **S**_*X*_.

Since the stoichiometric description counts the total amount of produced and consumed species by the reactions, a process **v** is represented by a vector in which its *i*-th coordinate **v**[*i*] specifies number of times (or the rate at which) $r_{i}\in R$ occurs.

In addition, the state of a reaction network is represented by a vector **x**_*t*_ of non-negative coordinates, where **x**_*t*_[*j*] counts the number (or concentration) of species of type *s*_*j*_ in the reaction network, *j* = 1, …, *m*, at time *t*. In discrete dynamics, we have that the state **x**_*t*+Δ*t*_(**v**) of the reaction network associated to a *current* state **x**_*t*_ and a process **v** occurring between the time-step *t* and *t* + Δ*t* is given by the following equation:

$$x_{t+\Delta t}\left(v\right)=x_{t}+Sv.$$ (1)

Equation 1 provides a formal description for the change of the number of species driven by a process **v**.

COT is concerned with processes that are self-producing in closed sub-networks. Such processes entail all possible forms of autopoietic organization of a reaction network and thus they represent the *cognitive domain* of the sub-network when viewed as a system (Varela, 1979; Maturana and Varela, 2012). Self-production is achieved by a process where all consumed species are produced by the reactions in it. Formally, let **v** be a non-null process. We say *X* is weak-self-maintaining with respect to **v** if and only if **x**_*t*+Δ*t*_[*j*] ≥**x**_*t*_[*j*], *j* = 1, …, *m*. If, additionally, **v** satisfies **v**[*i*] > 0 if and only if $r_{i}\in R_{X}$ , we say *X* self-maintaining.

For a weak-self-maintaining set *X*, there exists a process that induces non-negative production of all the species consumed by the process. However, such process might not execute all the reactions in $R_{X}$ . The latter is not useful for a realistic self-maintainance criteria because we expect that for every reaction that can happen then it should happen at some point. Hence, for self-maintaining sets, the process that leads to non-negative production is demanded to trigger every reaction in $R_{X}$ at some positive rate. Therefore, self-maintaining sets entail the parts of the reaction network where realistic self-sustainable processes, at a quantitative level of description can occur.

In continuous dynamics, the state vector is a function of time **x**(*t*) = (*x*_1_(*t*), …, *x*_*m*_(*t*)), where *x*_*j*_(*t*) encodes the number of species *s*_*j*_ at time *t*. In this case, Eq. 1 becomes

$$\dot{x\left(t\right)}=Sv\left(x\left(t\right)\right),$$ (2)

With initial conditions specified by **x**(*t*_0_), and **v** is a function of the state of the vector (and possibly other parameters).

Chemical Organization Theory (Dittrich and Di Fenizio, 2007) introduced the crucial notion of organization to link the stoichiometric analysis with the continuous dynamics.

Definition 1 *X* *is an organization if and only if* *X* *is closed and self-maintaining.*

Organizations entail a structural and stoichiometric form of persistence. From here, it is possible to link the notion of organization with the dynamics of a reaction network. Before doing so, a few definitions must be introduced:

Definition 2 *Let* $P\left(M\right)$ *be the power set of* M *,* *ϵ* *be a concentration threshold, and*

$$\phi \left(t\right):R_{\geq 0}^{m}\to P\left(M\right),\;x\left(t\right)\mapsto \phi \left(x\left(t\right)\right)\equiv \left\{s_{i}\in M:\;x_{i}\left(t\right)>\epsilon \right\}.$$ (3)

*For a state* $x\left(t\right)\in R_{\geq 0}^{m}$ *, the set* $\phi \left(x\left(t\right)\right)$ *is the* ***abstraction*** *of* **x**(*t*)*. For a given set of species* X⊆M *, a state* $x\left(t\right)\in R_{\geq \epsilon }^{m}$ *is an* ***instance*** *of* *X* *if and only if its abstraction equals* *X* *.*

The notions of abstraction encodes the set of species which are active enough (concentration above a threshold) to be considered active in the dynamics. An instance is the reverse notion, indicating whether or not a particular state has for abstract a certain set of a species. Both notions allow for connecting the representations of the reaction network with the dynamical system (2).

Organizations represent the abstractions of stable instances:

Theorem 1 *If* **x** *is a fixed-point of the ODE* (2)*, i.e.,* **Sv**(**x**) = **0** *, then the abstraction* *ϕ*(**x**) *is an organization* (Dittrich and Di Fenizio, 2007)*.*

Fixed points are the most simple stable behavior of a dynamical system (Strogatz, 2018). Theorem 1 provides a necessary condition for a set of species to form a sub-network with a stable behavior. In (Peter and Dittrich, 2011), the latter result is extended to most stable behaviors, including periodic orbits and limit cycles. In addition, various other studies explore the relations between organizations and stable dynamical behaviour in different kinds of systems (Peter et al., 2010; Kreyssig et al., 2012; Kreyssig et al., 2014; Veloz et al., 2014; Veloz, 2020; Rubin et al., 2021; Veloz and Flores, 2021).

### 3.3 Organizations as Emergent Goals

In the COT approach, we start from a set of relevant entities, which can be of any nature (physical, cognitive, economic, etc.), and from a set of rules that specify how combinations of these entities transform into new combinations. In this sense, the COT approach resembles various other system approaches based on inputs and outputs (Fishwick, 2007), and it is indeed mathematically equivalent to a Petri Net model (Reddy et al., 1993). However, in our approach the species and entities forming the reaction network do not represent a system and/or its environment, but a universe of transformative interactions. Therefore, no entity or structure within the reaction network is in advance assumed to be a system.

Since it is widely acknowledged that a system must hold some form of identity that allows to conceive it as an structurally independent entity within the universe of interactions (Varela, 1979), and that such independent identity must be stable enough so it can be subjected to observational procedures to be recognized as such (Goguen and Varela, 1979), we propose that an adequate notion of system is a subnetwork $\left(X,R_{X}\right)$ such that *X* is an organization (Veloz and Razeto-Barry, 2017a). Therefore, systems in our approach are conceived as organizations that *emerge from the universe of interactions specified by the reaction network*, similarly to what Maturana and Varela proposed in their theory of autopoiesis (Varela, 1979; Maturana and Varela, 2012; Razeto-Barry, 2012).

In consequence, our notion of system incorporates a basic form of emergent goal which is *to be a system*. The latter view is in accordance with the statement *the purpose of a system is what it does* or POSIWID, made famous by Stafford Beer in the early days of cybernetics (Beer, 2002). Since every goal must be reached and persist in order to be observed, and must be the result of the local actions that can possibly occur, we propose that beyond the fundamental notion of goal described above, different kinds of goals can be operationalized by giving further specifications of the feedback mechanisms that underlie the organization’s operation (Busseniers et al., 2021).

For example, note that when a self-maintaining process is applied to the reaction network none of the species decreases its amount (by definition). Hence, some species maintain their amount, i.e. they have production equal to zero, and other species increase their amount, i.e. they are overproduced as a positive feedback mechanism within the self-maintaining process. In particular, regular metabolic networks are expected to optimize the overproduction of biomass, and economic systems are expected to optimize the overproduction of money or value. Hence, the overproduction of one or many species, or some overproduction trade-off, can be easily specified, in operational terms, as a goal in the COT framework.

Another class of goal that can be easily operationalized is reaching or maintaining the occurrence of certain reactions or processes within certain levels. The latter implies that interactions tend to maintain some relative frequency, indicating that certain events remain happening within a certain range. The latter requires a the existence of negative feedback mechanism within the processes happening in the reaction network.

Another class of goals can be operationalized in terms of properties of a given module of the network. Namely, we might not want specify constrains for species or reactions, but for sub-networks of the organization. Recall that organizations form a hierarchy, thus an organization might contain several organizations, and such organizations contain other structures that are relevant for the self-maintaining behaviour (Veloz et al., 2018). In particular, COT is equipped with various notions for decomposing a reaction network into parts which are dynamically independent, meaning that the productive processes can be disentangled into groups which do not affect each other, and thus contain independent feedback mechanisms. This idea is particularly interesting because it allows to infer some aspects related to the evolution of the reaction network when it is perturbed (Veloz and Razeto-Barry, 2017b).

In addition to operationalizing the relation between feedback mechanisms and emergence, we can show how our operationalization is compatible with the definitions of emergence reviewed in section 2. Indeed, note that a system correspond to an organization. Thus, and differently to most emergence definitions, we do not need to specify further this concept.

Regarding Bonabeau and Dessalles definition (section 2.1), we can model a detector as any operator (functional) over the species state (and/or the process being applied to the system as explained above), and emergence is activated when such operator reaches a given value. For example, for the case of self-maintainance, the operator corresponds to the very self-maintainance condition, i.e, when **v** is such that **x**_**v**_[*j*] ≤ **x**[*j*] for all *j*, and **v**[*i*] > 0 for all *i*.

For Ronald and Sipper definition (section 2.2), we note that the reactions in a reaction network, which is the language *L*_1_ of the local interactions, corresponds to multiset operations, which are equivalent to *n*-dimensional vectors, i.e. linear algebraic operators of rank 1. Properties in COT rely instead on the sub-networks $\left(X,R_{X}\right)$ , which correspond to rank $|R_{X}|=k$ linear operators. Such language gap is similar to the change of expressivity that one encounters between propositional and first or higher order logics (Immerman, 1987). Indeed, all transitions of a reaction network can be written as propositional logic formulas, while the verification of self-maintainance of a particular process relies on the *for all* logical operator, as it demands a condition to be fulfilled for all the coordinates of the state vector, regardless of the size of the reaction network. Hence, the verification of self-maintainance entails a propositional to first order logic gap. Moreover, the identification of a self-maintaining reaction network requires to verify the *existence* of a flux vector holding the latter first order logical condition, so the property of *being an organization* corresponds to an even larger expressivity gap, between propositional and second order logics (Centler et al., 2008).

Regarding Baas’ definition (section 2.3), it is interesting to note that *S* ^2^ consists of the emergent organizations. Indeed, organizations appear as the result of the low level dynamics, i.e. the reactions, forming a landscape of stable reaction networks, where interactions are not any longer possible between species only, but between organizations. The emergence of a new layer of representation emerging from the level of reactions to the level of organizations is part of our ongoing research, but the core of this idea has been discussed previously in (Peter et al., 2010; Veloz et al., 2018).

Regarding Darley’s definition (section 2.4), it is possible to explain his definition by noting that in order to obtain the long-term dynamics of a reaction network it is necessary to run a simulation for enough time so we can confidently say that the network has passed the transient and settled in some particular attractor. However, COT allows to identify in an abstract way the possible long-term behaviours by means of linear programming computations which identify the self-maintaining sets (Centler et al., 2008). Hence, we could think of the algorithm that identifies organizations as the *deeper understanding* of the dynamics, and the dynamical simulation as the actual production of the emergent property.

Regarding Kubík’s definition (section 2.5), we are in partial disagreement with the idea that emergence can not be reached by the addition of behaviours of the parts, as the notion of organization relies on a linear algebraic operation, which is in ultimate terms the addition of vectors (Karp and Miller, 1969). However, there is an aspect of reaction networks that is compatible with the idea of non-additive behaviour. Namely, two organizations $X_{1},X_{2}\subset M$ have *synergy* when there exist a reaction in $R_{X_{1}\cup X_{2}}$ which is not in $R_{X_{1}}\cup R_{X_{2}}$ (Veloz et al., 2018). Therefore COT allows to observe Kubik’s kind of emergent behaviour, but such behaviour is non-additive from a local perspective of *X*_1_ and *X*_2_, while it remains additive within the perspective of the full reaction network (M,R) .

### 3.4 Towards the Modeling of Types of Emergence

Note that our notion of emergent goal, and the discussion of how it compares to the other definitions of emergence, assumes implicitly that we are in a non-changing environment, in the sense that the rules for the happening of the reactions remain static, and there is no addition or disappearance of species or reactions over time. Hence, our analysis is valid so far for type I emergence only (*see* Table 1). In order to provide an account of emergent goals for the other types of emergence we must allow for the possibility to dynamically modify the structure and behavioural rules of the reaction network. Thus, we are going to explain in more detail how we can frame such changes in COT and how that relate to other types of goal emergence.

In COT we identify three fundamentally different notions of context, which are used to identify three different types of change (Veloz and Razeto-Barry, 2017b). The first context corresponds the sub-network *X*, which determines what entities and interactions we consider, and thus the potentiality for a sub-network to be an organization. The second context operates within the first type of context, and specifies what processes **v** that are allowed to occur, and how they occur. The second context determines whether a sub-network is self-maintaining, i.e. if a self-maintaining process identified at the structural level is allowed by the rules for the happening of reactions (Peter et al., 2010), and thus an organization. The third context specifies the actual state of the reaction network in terms of how many species of each kind are at a particular time. Usually, the second type of context is a function of the third type of context, and of some other parameters of the reaction network such as kinetic rates. The latter specification rules the time-evolution of the reaction network from an initial state.

In the case of deterministic dynamics it is enough to know the initial state to derive all future states of the system when the second and third context are fixed. In stochastic dynamics it is also required to know what are the sources and extents of stochastic variation to provide a complete account of the evolution of the state over time. Note that in the third type of context is where the usual notion of perturbation of dynamical system analysis lies on (Strogatz, 2018).

Thus, when the three contexts are determined, we are able to simulate the system and know if the system is able reach the emergent goal of becoming persistent in time. Furthermore, we can check if the dynamics hold further behavioral properties beyond persistence such as resistance to state perturbations (stability analysis), or optimization of a certain functional related to the state and process values (Fell, 1992; Fishwick, 2007). Therefore, most properties pertinent to the analysis of dynamical systems apply to type I emergence according to (Fromm, 2005).

Interestingly, the remaining three types of emergence developed by (Fromm, 2005) can be directly related to changes of the first and second context described above. Namely, emergence of type II is described as flexible roles, which in our setting corresponds to changing the second type of context, i.e. changes in the way processes occur and thus in the way species play different roles within the self-productive dynamics, in the absence of changes at the first type of context (structural changes). For example, for two self-maintaining process vectors, a given species can be and be not overproduced respectively. The latter has deep impact in the self-maintaining dynamics and in relation to the production of other species (Veloz et al., 2021). Emergence of type III corresponds to the appearance and disappearance of roles. For a role to appear or disappear we require a change in the structure of reactions, with the possible appearance of new species, or the disappearance of current ones. This is exactly what it is specified by the first type of context specified above. In particular, the decomposition theorem in COT can be applied to understand what parts of the reaction network will be affected by such structural perturbation and whether or not it will remain as an organization after the structural perturbation (Veloz and Razeto-Barry, 2017b). A better picture of the latter is to think of sustained changes of first and second type over time, emulating ontogeny or systems with evolvabilty (Valiant, 2009), so the reaction network will constantly suffer structural and behavioural changes, while remaining as an organization, and thus without loosing its identity (Maturana and Varela, 2012). Finally, emergence of type IV is the opening of a whole new world of roles, which in our case corresponds to a strong combination of types of change of first and second kind so that the reaction network after change becomes a completely different structure. This correspond to phenomena such as major transitions in evolution [?]nd is understood in

## 4 Conclusion

With the aim of providing an operational account of the notion of emergent goal, we reviewed the most remarkable attempts to operationally define emergence, and found that, even though the notion of emergent property is well captured (either by means of representational reduction of complexity, surprise, change of language, new observational mechanisms, etc.), they assume in advance the existence of a system that is going to be observed or represented prior and post the happening of the emergent property, and thus lack an operational description of how systems become as such. The latter makes difficult to operationalize the very configuration of a system as a form of goal emergence. For this reason we started with an operationalization of the notion of becoming a system as an emergent goal. Particularly, COT allows to determine systems as emergent autopoietic processes operating on a reaction network, so-called organizations, and following the POSIWID approach (Beer, 2002) we consider the notion of organization to be the basis of emergent goals. This idea simplifies in various ways the problems encountered in understanding both the notion of emergence and of goal. Particularly, the problem of observation is reduced to the ability of the system to persist, and the problem of specifying the goal is reduced to our ability to define a certain feature (that can be operationalized as a functional over the reaction networks dynamics) in the actual system. We next explained how the types of change that a reaction network can undergo allow to operationalize the different forms of emergence described in the literature. The operational basis of COT that allows to link types of change of a reaction network with its structural stability and thus with potential emergent goals, opens various venues for future work. For example, it is necessary to advance further on the relation between modular structural properties of the reaction network and its behaviour (Veloz and Razeto-Barry, 2017b; Veloz et al., 2021), as well as establishing concrete models of the specific feedback mechanisms that are specified in the more complex forms of emergence (Fromm, 2005). As a simple example of the latter, the typical negative feedback mechanism found in Lotka-Volterra systems can be modeled by three reactions as follows:

$$r_{1}:\to s_{1},\;r_{2}:s_{1}+s_{2}\to 2s_{2},\;r_{3}:s_{2}\to .$$

Indeed, *s*_2_ will tend to grow fast until it is close to deplete *s*_1_ and then it will stabilize in an oscillatory way towards a stable regime coupled with the way *s*_1_ enters in the system.

Therefore, the creation and fixation of feedback mechanisms in environments where multiple entities not only interact in different ways, but also entities and interactions can disappear, be modified, or appear, can be operationally identified by computing the organizations of a changing reaction network where reactions and species appear and disappear (Busseniers et al., 2021).

We believe that reaction network modeling approach to systems might be useful to formalize several not-well understood notions in systems theory and of complex systems in general. The latter will contribute to develop methods for interdisiciplinary and transdisciplinary identification, framing, and solutions of this century problems.

## Data Availability

The original contributions presented in the study are included in the article/supplementary material, further inquiries can be directed to the corresponding authors.
